# Broken tibial nail extraction: a useful technique

**DOI:** 10.1308/rcsann.2023.0025

**Published:** 2024-03-06

**Authors:** S Walters, A Trompeter

**Affiliations:** St George’s University Hospital, UK

## Background

A broken tibial nail can be challenging, generally requiring removal before proceeding with further fixation. Extracting the distal segment poses difficulties, and there are several minimally invasive techniques to either hook the distal end of the nail,^1,2^ or create an interference fit,^3,4^ and specific instruments such as nail removal hooks now exist for this purpose. When unsuccessful, more invasive techniques, such as removal of part of the cortex for access,^5^ can be employed but ideally surgeons should be aware of numerous minimally invasive options to avoid this.

## Technique

**Figure 1:**
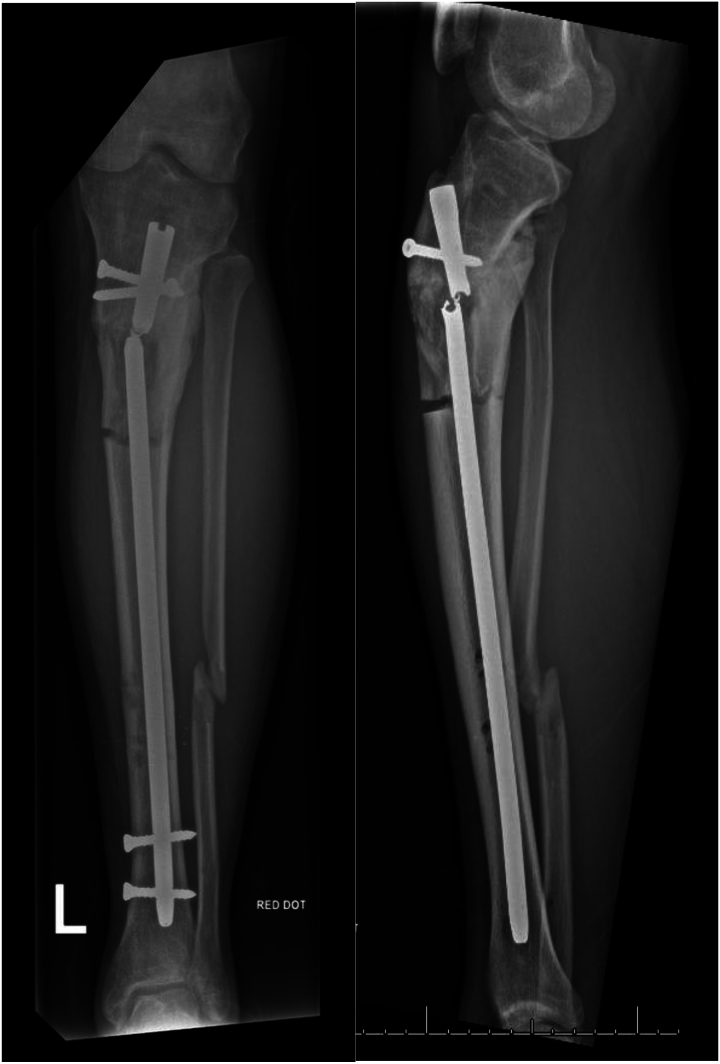
Broken intramedullary nail with tibial non-union

**Figure 2:**
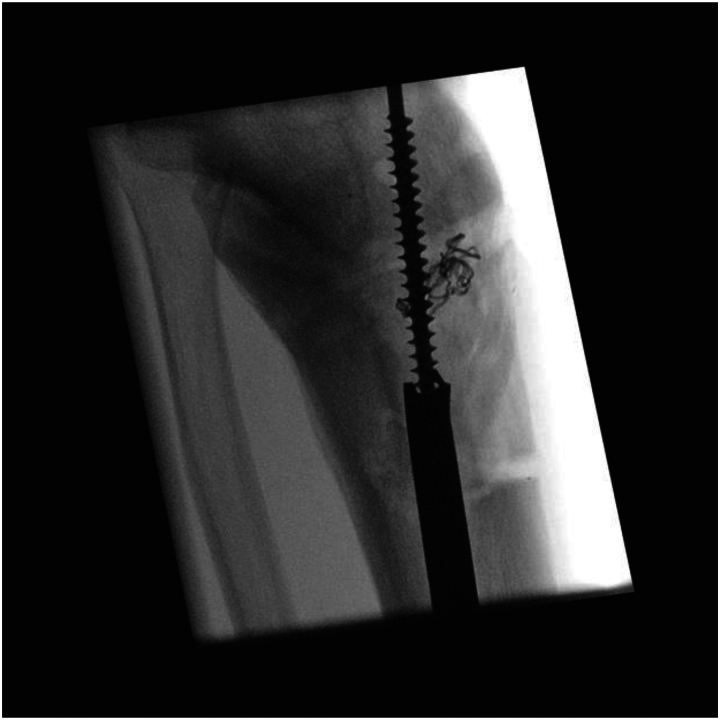
Tap (6.5mm) engaged inside broken distal segment of tibial nail

**Figure 3:**
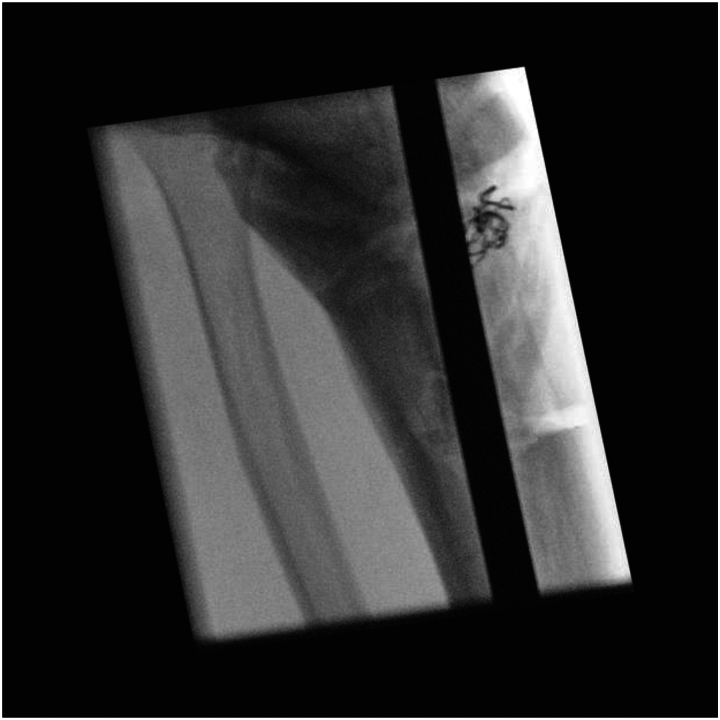
Intramedullary nail pulled out using the tap

For a broken tibial nail (Figure 1), the first steps are to remove the locking bolts and proximal nail segment. Removal of the distal segment can be attempted using extraction instruments, but if conventional methods are unsuccessful, this technique can be utilised. A 6.5mm tap from a large fragment set (e.g. Stryker Basic Fragment) is introduced into the nail segment on a handle, using image intensifier guidance. This is carefully advanced and rotated to engage the threads of the tap into the nail (stainless steel instrument with greater hardness than a titanium nail) (Figure 2). Once purchase is achieved, the tap is used to extract the nail (Figure 3). Care must be taken to avoid bending or excessive torque on the tap to avoid breakage, and usage of this technique is not supported by the manufacturer.

## Discussion

This simple technique is a useful addition to any surgeon’s inventory for removing a challenging broken intramedullary nail.

## References

[1] Levine JW, Georgiadis GM. Removal of a broken cannulated tibial nail: a simple intramedullary technique. *J Orthop Trauma* 2004; **18**: 247–249. 15087971 10.1097/00005131-200404000-00011

[2] Marwan M, Ibrahim M. Simple method for retrieval of distal segment of the broken interlocking intramedullary nail. *Injury* 1999; **30**: 333–335. 10505126 10.1016/s0020-1383(99)00092-3

[3] Sivananthan KS, Raveendran K, Kumar T, Sivananthan S. A simple method for removal of a broken intramedullary nail. *Injury* 2000; **31**: 433–434. 10831741 10.1016/s0020-1383(00)00015-2

[4] Steinberg EL, Luger E, Menahem A, Helfet DL. Removal of a broken distal closed section intramedullary nail: report of a case using a simple method. *J Orthop Trauma* 2004; **18**: 233–235. 15087967 10.1097/00005131-200404000-00007

[5] Somerville CM, Hanschell H, Tofighi M, Lahoti O. A novel surgical technique for extraction of a firmly integrated broken intramedullary nail. *Strateg Trauma Limb Reconstr* 2022; **17**: 55–58. 10.5005/jp-journals-10080-1550 PMC9166259 35734035

